# Mr. Twitch, on the Swine Pox

**Published:** 1800-03

**Authors:** J. Twitch

**Affiliations:** Salisbury


### *To the Editors of the Medical and Phyfical Journal.*

GENTLEMEN,

HAVING had an opportunity of examining the bodies of pigs, that have died of a difeafe which is very common to thofe animals, and which frequently proves fatal; and as no one has hitherto given a clear defcription of the morbid appearances of the body after death, or pointed out an effectual method of treating the difeafe, I thought the following obfervations might be ufeful to the public, becaufe I truft they will fuggeft a mode of treatment that will be found generally fuccefsful; and, confequently, I prefumed that there would be no impropriety in requefting you to communicate them through the medium of your ufeful Journal.

The difeafe alluded to is, by fome, called the Swine Pox; in fome places it is called the heavings; in others, the meafles; in fhort, it has a great variety of names. It is the moft common difeafe among pigs, and appears to arife frequently from contagion. On the firft attack, the animal appears to be fick, and refufes his food; he becomes heavy, and unwilling to move, and there is generally a weak cough, attended with a convulfive twitching of the abdominal mufcles, or flanks; there is generally a fcaly kind of eruption on the body alfo. As the difeafe advances, the voice acquires a peculiar fhrill tone, and becomes extremely weak; the pupils of the eyes are fometimes dilated, and the retina appears to have loft, in a great meafure, its fenfibility to light. Sometimes the animal appears to be delirious, running againft the wall, or any thing that may be in its way. On examining the body after death, the ftomach was found highly inflamed, and there were feveral perfectly black fpots on its internal furface. The inteftines were alfo highly inflamed on both furfaces, and in fome parts had nearly approached to a gangrenous ftate; the inflammation was more confiderable in the fmall than in the large inteftines. The lungs were likewife inflamed, and adhered in feveral parts to the infide of the ribs. The bronchiæ were loaded with infpiffated mucus, and the membrane which lines them was confiderably inflamed. The liver was healthy, but the gall bladder appeared to be diftended with bile, though no obftruction could

 be be difcovered in the duct. The kidneys, fpleen, and bladder, were in a natural ftate, but the urethra was inflamed. The veffels of the brain were confiderably diftended, particularly on its furface; and the plexus choroides was much enlarged. There was no fluid in the ventricles.

From thefe appearances, it is very evident that the difeafe depends on general inflammation, and that the variation fometimes obferved on the fymptoms is occafioned by the inflammation being more confiderable in one part than another. With regard to the remote caufes of the difeafe, it is not improbable that the infectious matter is originally produced by keeping thefe animals in too clofe a place, and neglecting cleanlinefs; but in fome inftances the difeafe appears to have arifen fpontaneoufly. The following is the mode of treatment I would recommend: As foon as the difeafe is obferved, the animal is to be bled; this is commonly done by cutting off part of the ear or tail, by which a fufficient quantity of blood cannot be procured; but there are two arteries in the roof of the mouth, which may be eafily opened with a gum lancet, or a common fleam. Should the bleeding continue longer than is neceffary, it may be ftopped by placing a pledget of tow on the orifice, and confining it by means of tape brought round the nofe; but it is proper to draw a *confiderable* quantity of blood in this difeafe. Two ounces of fallad oil are to be given, though Caftor oil would, probably, be more efficacious; and clyfters of water gruel, with fweet oil, will be found very ufeful. If thefe remedies be applied at the commencement of the difeafe, I think they will be generally fuccefsful; and in many cafes, it is probable, that a proper bleeding, alone, at the firft attack, will fpeedily remove the difeafe. But when it has been neglected, or improperly treated at firft, the cure is by no means fo certain, and it will be neceffary to make an incifion under each ear, and to fill the wound with tow, moiftened with oil of turpentine. It will be found ufeful alfo to raife inflammation on the abdomen and cheft, by means of blifters, or the actual cautery, if thofe parts are not covered with the eruption.

Therefore, it is of the greateft confequence to attack the difeafe at its firft appearance, for in this will our fuccefs in a great meafure depend.

I have the honour to be,

GENTLEMEN,

Your moft obedient fervant,

J. TWITCH.

